# Oxalic acid degradation in wood-rotting fungi. Searching for a new source of oxalate oxidase

**DOI:** 10.1007/s11274-022-03449-4

**Published:** 2022-11-16

**Authors:** Marcin Grąz, Marta Ruminowicz-Stefaniuk, Anna Jarosz-Wilkołazka

**Affiliations:** Department of Biochemistry and Biotechnology, Institute of Biological Sciences, Maria Curie-Skłodowska University, 20-033 Lublin, Poland

**Keywords:** Oxalate, Oxalate oxidase, Oxalate decarboxylase, Fungi, Low molecular weight compound, Wood

## Abstract

Oxalate oxidase (EC 1.2.3.4) is an oxalate-decomposing enzyme predominantly found in plants but also described in basidiomycete fungi. In this study, we investigated 23 fungi to determine their capability of oxalic acid degradation. After analyzing their secretomes for the products of the oxalic acid-degrading enzyme activity, three groups were distinguished among the fungi studied. The first group comprised nine fungi classified as oxalate oxidase producers, as their secretome pattern revealed an increase in the hydrogen peroxide concentration, no formic acid, and a reduction in the oxalic acid content. The second group of fungi comprised eight fungi described as oxalate decarboxylase producers characterized by an increase in the formic acid level associated with a decrease in the oxalate content in their secretomes. In the secretomes of the third group of six fungi, no increase in formic acid or hydrogen peroxide contents was observed but a decline in the oxalate level was found. The intracellular activity of OXO in the mycelia of *Schizophyllum commune*, *Trametes hirsuta*, *Gloeophyllum trabeum*, *Abortiporus biennis*, *Cerrena unicolor*, *Ceriosporopsis mediosetigera*, *Trametes sanguinea*, *Ceriporiopsis subvermispora*, and *Laetiporus sulphureus* was confirmed by a spectrophotometric assay.

## Introduction

Oxalic acid is the most common organic acid secreted by a majority of fungi (Jarosz-Wilkołazka and Grąz 2006; Shimada et al. 1997). It plays multiple roles in fungal physiology and exerts an important impact on environmental processes, e.g. nutrient availability, weathering, or competition between organisms (Gadd 2007). For example, oxalate has a large effect on the availability of phosphorous and calcium and is thus linked to the weathering of soil minerals and the precipitation of insoluble metal oxalates (Gadd et al. 2014; Jarosz-Wilkołazka and Grąz 2006; Dutton and Evans 1996). Basidiomycota fungi are very efficient wood degraders (Janusz et al. 2017). Oxalic acid is classified as a low molecular weight compound (LMWC) involved in lignocellulose biodegradation. This low molecular weight fraction of the fungal secretome is involved in all stages of wood decay and comprises such compounds as reactive oxygen species, aromatic compounds, transition metal coordination complexes, peptides, and organic acids (Janusz et al. 2017; Plassard and Fransson 2009). In particular, LMWC are involved in the initial stages of wood biodegradation due to the fact that an intact wood cell wall requires preparation for the enzymatic step of degradation (Nousiainen et al. 2014; Baldrian and Valaskova 2008). Oxalic acid is an important factor in the process of chelation of Fe^3+^ ions or heavy metal sequestration. It also takes part in free radical formation related to Fenton reaction (Zhu et al. 2016; Gadd 2007; Aguiar et al. 2006). Oxalate also serves as a donor or acceptor of electrons, a metal chelator involved in manganese-dependent peroxidase (MnP) catalytic cycle, or an osmotic and pH regulator (Hofrichter 2002; Munir et al. 2001). The concentration of oxalate in the fungal environment is controlled due to the toxic effect of oxalates and their influence on fungal enzyme activities (Presley et al. 2018; Hastrup et al. 2012; Shimada et al. 1997). Given the role of oxalates in the degradation of the ligninocellulose complex, learning about oxalic acid metabolism in fungi can help to develop effective strategies for wood decomposition or protecting wood from fungal decay. It seems to be important due to potential application of fungal ability to efficiently lignocellulose complex conversion in e.g. biofuel production (Saini and Sharma 2021) or to reduce the significant economic costs caused by wood-degrading fungi (Schmidt 2007). So far, four enzymatic activities for the decomposition of oxalic acid have been classified and attributed to particular groups of organisms: (1) the decarboxylation of oxalic acid catalyzed by oxalate decarboxylase (EC 4.1.1.2), typical for fungi, leading to the formation of formic acid and carbon dioxide, (2) the decarboxylation of activated oxalic acid molecules (oxalyl-CoA) catalyzed by oxalyl-Co decarboxylase (EC 4.1.1.8) yielding formyl-CoA and carbon dioxide, (3) the oxidation by thiamine pyrophosphate (TPP)-dependent oxalate oxidoreductase (OOR) generating two CO_2_ molecules and two low-potential electrons, both typical for bacteria, and (4) the oxidation of oxalic acid catalyzed by oxalate oxidase (EC 1.2.3.4), which is widespread in plants and leads to formation of carbon dioxide and hydrogen peroxide (Gibson et al. 2016; Mäkelä et al. 2010; Svedruzic et al. 2005). There are exceptions to this rigid division. The oxalyl-Co decarboxylase activity was proposed in *Arabidopsis thaliana* for oxalic acid degradation (Foster et al. 2012). An oxidative pathway of oxalic acid decomposition by fungi via the action of fungal oxalate oxidase (OXO, EC 1.2.3.4) was also confirmed. Such activity was detected in *Ceriporiopsis subvermispora* (Escutia et al. 2005; Aguilar et al. 1999) and *Abortiporus biennis* (Grąz et al. 2016, 2009). Effective decomposition of oxalic acid can provide some benefits in diagnostic, agricultural, and medical applications. Oxalate oxidase (OXO) can be applied as a tool in diagnostic kits in oxalate concentration assays, as an antifungal factor against plant pathogenic fungi, or in efforts to improve the quality of edible plants (Pfau et al. 2020; Kumar et al. 2019; Qi et al. 2017; Heller and Witt-Geiges 2013).

The purpose of the present work is to verify the mode of oxalic acid decomposition in cultures of different wood-rotting fungi to find whether the oxidative pathway of oxalate decomposition can be more widespread than is currently thought. It is important and may lead to the isolation of new enzyme for oxalate decomposition with potentially better biochemical properties. They can find biotechnological applications like prevention of kidney stone formation and control of plant-pathogenic fungi. The excess of oxalate in the human diet may promote kidney stone formation and urinary tract disorders (Buysschaert et al. 2020). Oxalate-decomposing enzymes can also be an antifungal factor against pathogenic fungi which use oxalic acid as a virulence factor in plant tissue disorders (Heller and Witt-Geiges 2013).

## Materials and methods

### Fungal strains and culture conditions

All fungal strains used in the study were obtained from Fungal Collection (FCL) of the Department of Biochemistry and Biotechnology, Maria Curie-Skłodowska University, Lublin, Poland (Table 1). Stock cultures were maintained on 2% (m/v) malt agar at 4 °C. Fungal strains were precultured on 2% (m/v) malt extract agar for 1 week at 25 °C. The experiment were performed in 100 mL Erlenmeyer flasks using 50 mL liquid medium containing glucose (10 g L^−1^) and potato extract (4 g L^−1^). The inoculated flasks were stationary incubated at 25 °C. On the day 7 of culture, oxalic acid (10 mM final concentration in culture) was added sterilely to induce oxalic acid catabolism enzymes. The cultivation medium was collected in the 7, 8 and 9 day of cultivation and tested for oxalic acid, formic acid and hydrogen peroxide.

**Table 1:** List of the tested fungi with appropriate numbers in the Fungal Collection (FCL) of the Department of Biochemistry and Biotechnology UMCS

Species	Fungal collection number
*Abortiporus biennis*	123
*Agrocybe aegerita*	267
*Bjerkandera fumosa*	137
*Ceriosporopsis mediosetigera*	150
*Ceriporiopsis subvermispora*	273
*Cerrena unicolor*	139
*Flammulina velutipes*	68
*Fomes fomentarius*	25
*Fomitopsis pinicola*	282
*Ganoderma lucidum*	188
*Gloeophyllum odoratum*	124
*Gloeophyllum trabeum*	83
*Laetiporus sulphureus*	331
*Nematoloma frowardii*	275
*Phlebia radiata*	99
*Piptoporus betulinus*	307
*Pleurotus ostreatus*	103
*Pleurotus pulmonarius*	127
*Pleurotus sajor-caju*	237
*Schizophyllum commune*	12
*Trametes hirsuta*	19
*Trametes sanguinea*	199
*Trametes versicolor*	7

### Determination of oxalic and formic acids

The concentration of organic acids in the fungal cultures was monitored by capillary electrophoresis using an Agilent 7100 Capillary Electrophoresis System equipped with a DAD detector. The separation was carried out using a fused silica capillary 50 µm ID with a 50 cm length to the detection window. The voltage applied was − 25 kV and the capillary temperature was maintained at 15 °C. Samples were injected hydrodynamically for 5 s. (50 mbar) and organic acid was detected by indirect UV detection at a wavelength of 350 nm (bandwidth 20 nm) and a reference wavelength of 230 nm (bandwidth 10 nm). The buffer solution was freshly prepared every day by dissolving phthalic acid (5 mM), cetyltrimethylammonium bromide (CTAB, 0.26 mM), and methanol (0.5% v/v) in MiliQ water (Chen et al. 1997). Peak identification was done by spiking with commercially available formic and oxalic acids.

### Determination of hydrogen peroxide concentration 

Hydrogen peroxide content was determined by the Co(II) catalyzed oxidation of luminol. The solutions of luminol and Co(II) were prepared according to Pérez and Rubio 2006.

The reaction mixture contained 0.1 mL of sample and 1 mL of a reagent solutions of luminol and Co(II). The emitted photons were counted with the luminometer (Lumat LB 9507, Berthold. The formation of hydrogen peroxide was compared with the calibration curve and expressed in micromoles.

### Oxalate oxidase (OXO) activity assay 

The standard assay of this enzyme (Aguilar et al. 1999) was based on the measurement of enzymatically generated hydrogen peroxide. The reaction mixture contained 0.3 mL of 20 mM oxalic acid in 0.05 M succinate buffer, pH 3.5, and 0.2 mL of the enzyme. Reaction mixture was incubated for 15 min at 40 °C, than 0.45 mL of 0.2 mM phenol red solution and 0.05 mL of horseradish peroxidase (6.25 U) in 0.05 M succinate buffer, pH 3.5, were added. After 15 min incubation at 30 °C, 0.1 mL of 5 M NaOH was added and the absorbance at 610 nm was measured. A standard curve was used to calculate the amount of H_2_O_2_ generated during the OXO reaction. One enzyme unit was defined as the amount of enzyme required to produce 1 µM of H_2_O_2_ per minute, under standard assay conditions.

## Results

### Secretion of hydrogen peroxide into the culture media by fungi after oxalic acid addition

**Fig. 1:**
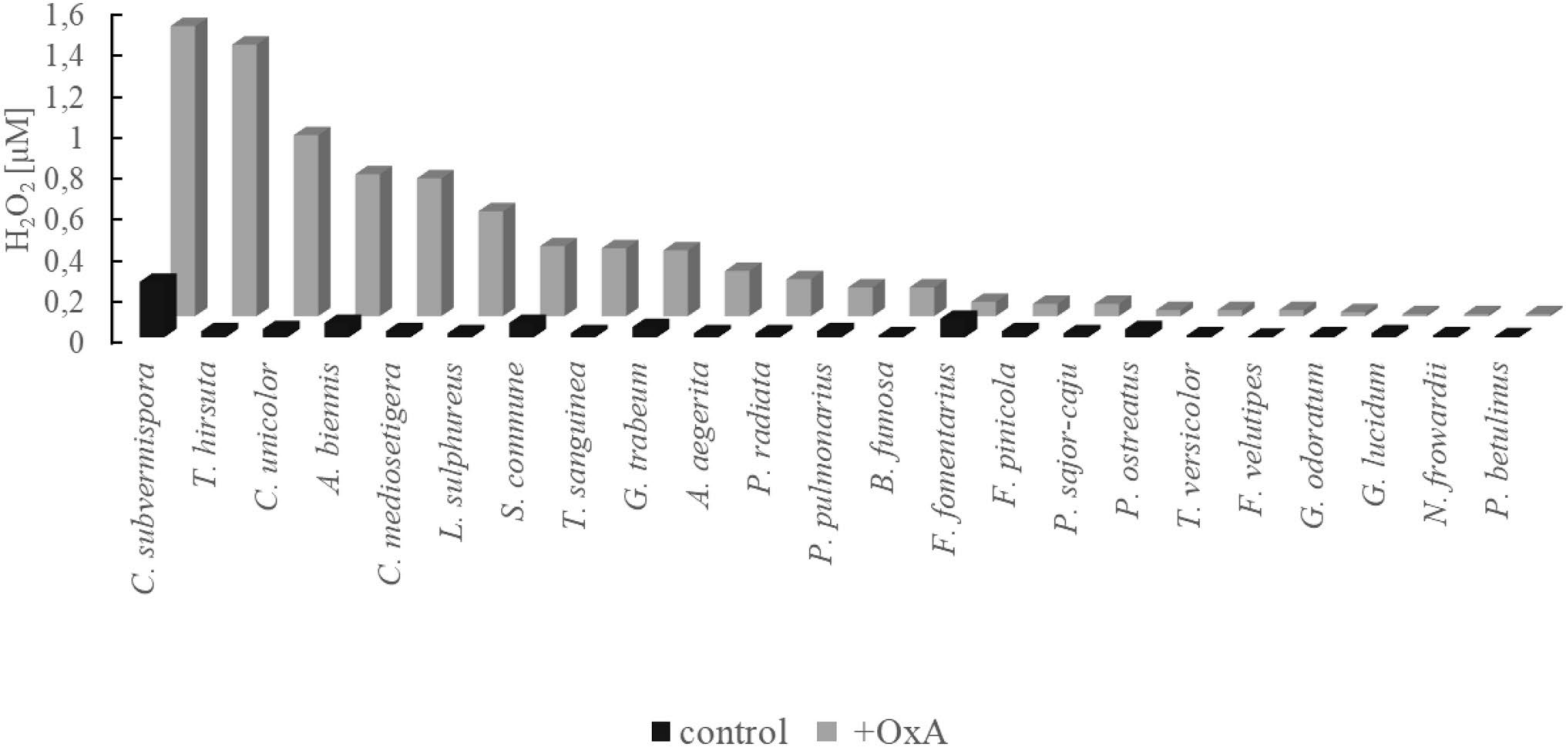
Hydrogen peroxide concentration in fungal cultures after 24 h of oxalic acid addition for the induction of oxalate-decomposing enzymes (+ OxA) *versus* non-induced cultures (control)

The presence of hydrogen peroxide in the fungal secretome may indicate metabolism of oxalic acid via oxalate oxidase; therefore, the H_2_O_2_ content was determined in the fungal cultures. Figure 1 presents the concentration of hydrogen peroxide detected in the control cultures of the fungi and in the cultures after 24 h of oxalic acid addition. We observed that the addition of oxalic acid stimulated a substantial increase in the content of hydrogen peroxide in some fungal cultures (Fig. 1). As presented in Fig. 1, we observed a sharp increase in the concentration of hydrogen peroxide in some cultures, e.g. in *C. subvermispora*, *T. hirsuta*, *C. unicolor*, *A. biennis*, *C. mediosetigera*, *L. sulphureus*, *S. commune*, *T. sanguinea*, *G. trabeum*, *A. aegerita*, *P. radiata*, *P. pulmonarius*, and *B. fumosa*. In these fungi, the detected hydrogen peroxide concentration ranged from 0.14 µM to 1.4 µM, and the differences in the hydrogen peroxide content between the oxalic acid-induced cultures and the control without oxalic acid addition were high, i.e. the content was from 5 to 44 times higher in the induced cultures. In the rest of the tested fungal secretomes (*F. fomentarius*, *F. pinicola*, *P. sajor-caju*, *P. ostreatus*, *T. versicolor*, *F. velutipes*, *G. odoratum*, *G. lucidum, N. frowardii* and *P. betulinus*), the hydrogen peroxide concentration was lower than 0.1 µM both in the oxalic acid-induced and non-amended cultures. The highest concentration of hydrogen peroxide was detected in the cultures of *C. subvermispora* and *T. hirsuta*, i.e. 1.4 µM and 1.3 µM, respectively. The *A. biennis* fungal culture reached a level of 0.7 µM of hydrogen peroxide in comparison to the content of 0.07 µM in the non-induced cultures of this fungus. The concentration of hydrogen peroxide in the oxalic acid non-induced cultures oscillated around values lower than 0.1 µM, with the exception of *C. subvermispora*, where it reached 0.27 µM. The content of hydrogen peroxide in the *F. fomentarius* cultures was comparable in both culture variants.

### Monitoring fungal secretomes for products of enzyme activities involved in oxalic acid degradation

**Fig. 2:**
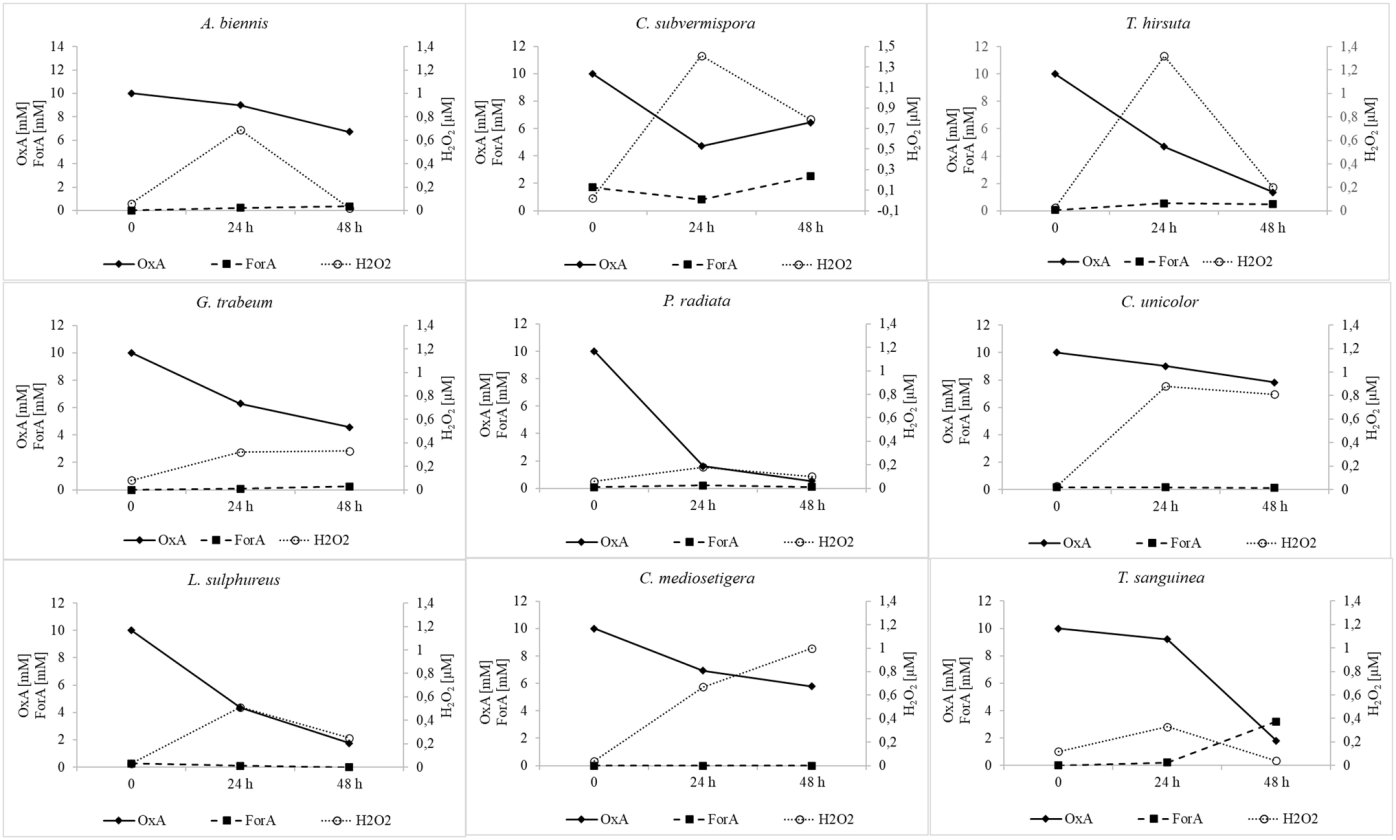
Secretome pattern of oxalic acid (OxA), formic acid (ForA), and hydrogen peroxide at 24 and 48 h after oxalic acid addition in fungal strains classified as oxalate oxidase producers, 0 – time of OxA addition

**Fig. 3:**
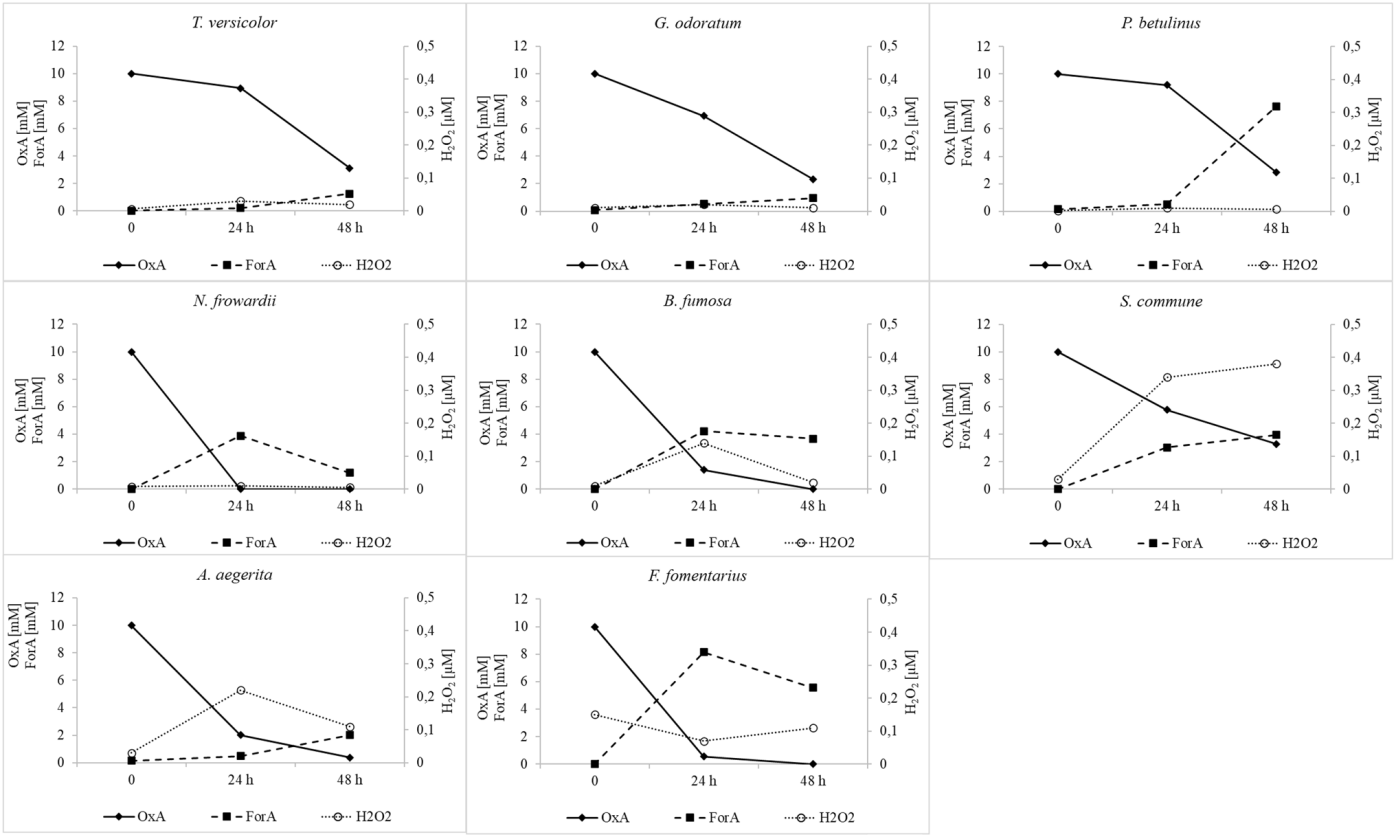
Secretome pattern of oxalic acid (OxA), formic acid (ForA), and hydrogen peroxide after 24 and 48 h of induction of the cultures by oxalic acid addition in fungal strains classified as oxalate decarboxylase producers, 0 – time of OxA addition

**Fig. 4:**
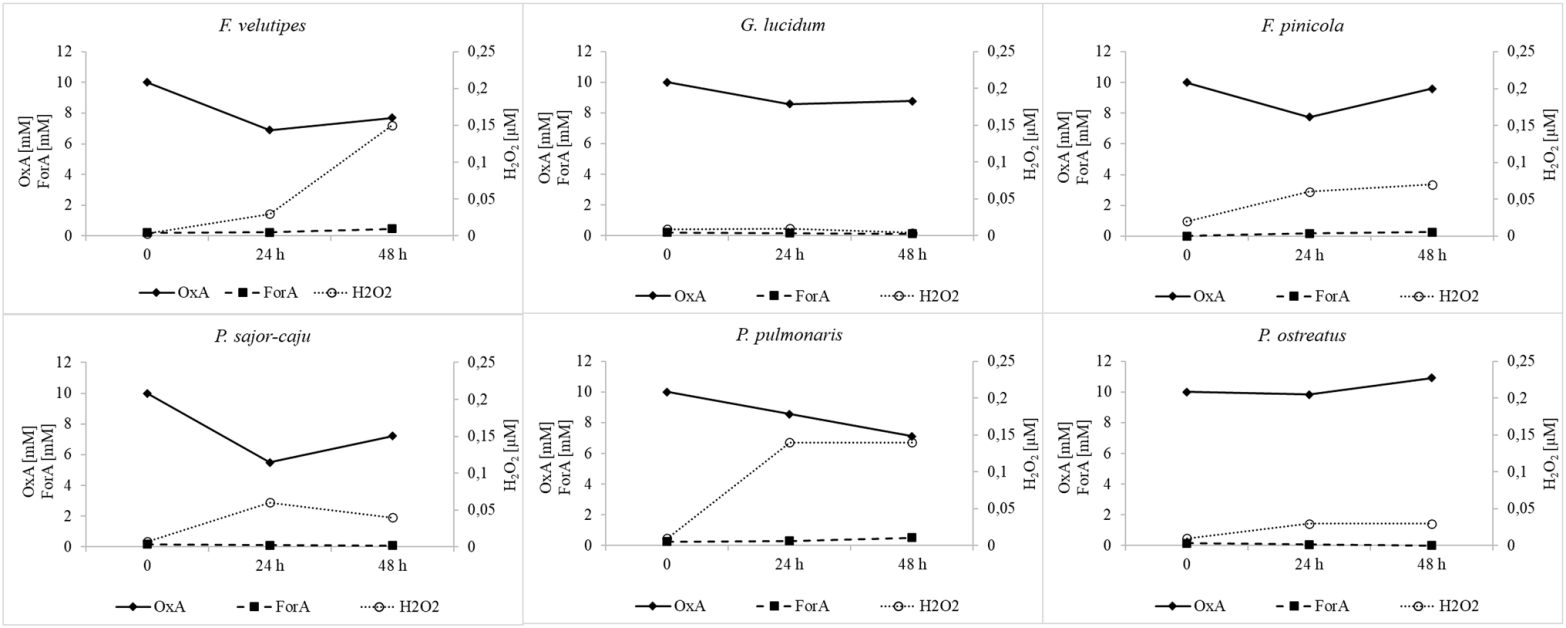
Secretome pattern of oxalic acid (OxA), formic acids (ForA), and hydrogen peroxide after 24 and 48 h of induction of the cultures by oxalic acid addition in fungal strains classified as oxalate decarboxylase or oxalate oxidase non-producers, 0 – time of OxA addition

To establish more precisely the oxalate degradation pathway in the tested fungi, the concentration of oxalic acid and changes in the formic acid level were monitored. Depending on the enzymes involved in oxalic acid degradation in fungi, two main products are expected to be found in fungal secretomes: formic acid or hydrogen peroxide. To demonstrate the pathway of oxalic acid degradation in the tested fungal cultures, the concentrations of oxalic and formic acids were monitored as well as the hydrogen peroxide content. As a result of monitoring the secretomes of the tested fungi for the oxalic acid, formic acid, and hydrogen peroxide content, the three main groups were identified. The first group (Fig. 2) comprised fungi with potential oxalate oxidase activity, and the following strains were classified into this group: *Abortiporus biennis*, *Ceriporiopsis subvermispora*, *Trametes hirsuta*, *Gloeophyllum trabeum*, *Phlebia radiata*, *Cerrena unicolor*, *Laetiporus sulphureus*, *Ceriporiopsis mediosetigera*, and *Trametes sanguinea*. These fungi were characterized by a drop in the oxalate concentration during two days of testing and a constant concentration of formic acid or a relatively insignificant increase in its content, as in the case of *T. sanguinea* and *C. subvermispora*. The main important feature observed was the significant increase in the hydrogen peroxide content in the tested media over the two days of the observation. Except for *P. radiata*, where the hydrogen peroxide concentration reached a maximum of 0.18 µM, in all secretomes of fungal strains classified to this group, hydrogen peroxide detected exceeded 0.5 µM reaching a value even above 1 µM, as in the case of *T. hirsuta* and *C. subvermispora*. The second group (Fig. 3) comprised fungal strains that accumulated formic acid in their culture media after the addition of exogenous oxalic acid. This may indicate the oxalate decarboxylase activity decomposing oxalic acid into formic acid and carbon dioxide. The following fungi were classified into this group: *Trametes versicolor*, *Gloeophyllum odoratum*, *Piptoporus betulinus*, *Nematoloma frowardii*, *Bjerkandera fumosa*, *Schizophyllum commune*, *Agrocybe aegerita*, and *Fomes fomentarius*. In all fungal cultures in the second group, the decline in the oxalate content was associated with an increasing formic acid concentration and a low level of hydrogen peroxide, whose concentration was below 0.25 µM. The exception was *S. commune*, where the increase in the formate content was observed in the presence of the increasing (up to 0.4 µM) level of hydrogen peroxide on cultivation day 9. The third group (Fig. 4) identified in the study comprised fungi exhibiting no significant increase in the contents of formic acid and hydrogen peroxide. The concentration of oxalic acid declined, but the content of oxalate in the case of *P. ostreatus* was not changed during the observation. This group included *Flammulina velutipes*, *Ganoderma lucidum*, *Fomis fomitopsis*, *Pleurotus sajor-caju*, *Pleurotus pulmoris*, and *Pleurotus ostreatus*.

### Oxalate oxidase activity in selected fungi

**Fig. 5:**
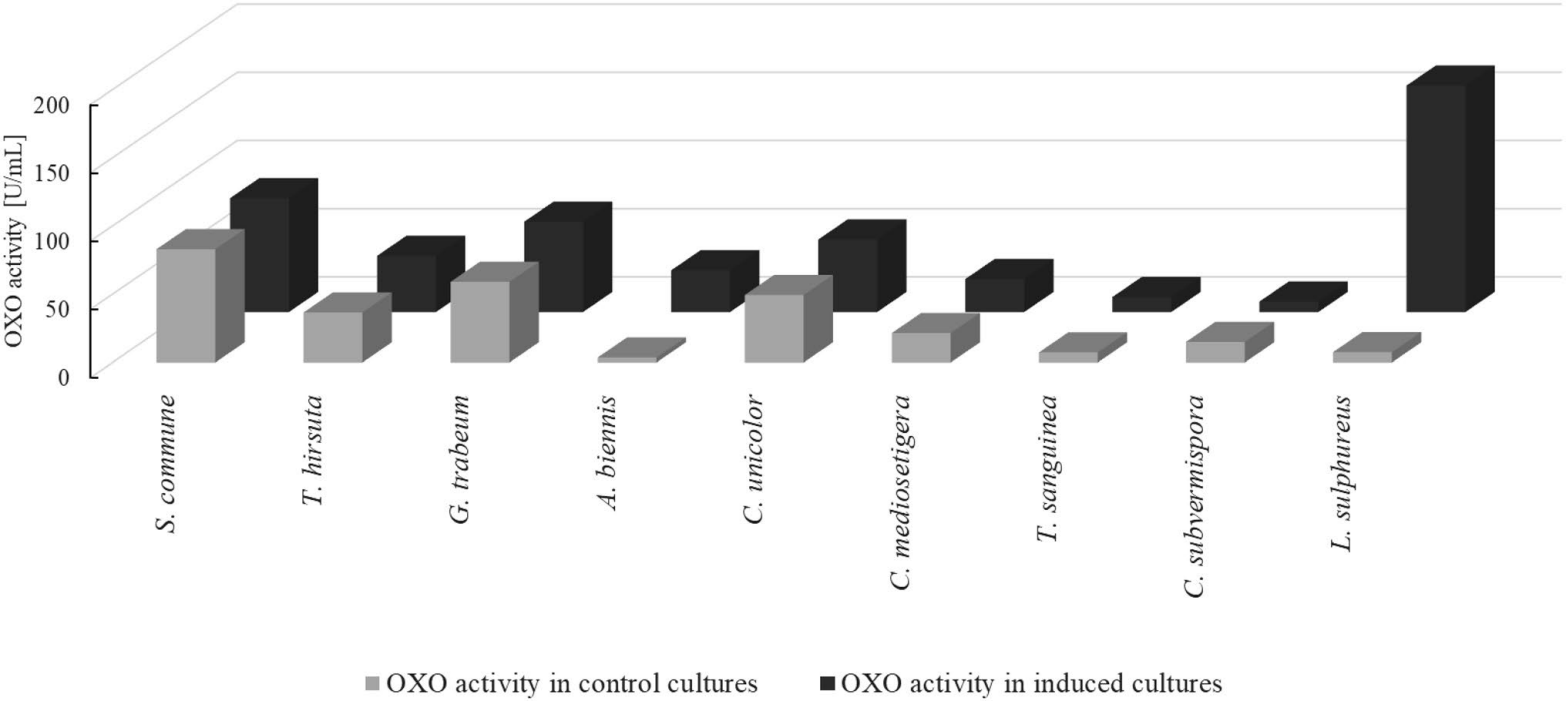
Intracellular oxalate oxidase activity of selected fungi in oxalic acid induced and non-induced cultures

Based on the observation of the composition of the products of enzymatic oxalic acid degradation in the secretomes, the mycelia of fungi classified into the first group and *S. commune* from the second group were selected for the study of intracellular OXO activity. *S. commune* was selected due to the increasing content of both formic acid and hydrogen peroxide in their secretome. The activity was measured in the mycelia after 24 h of oxalic acid addition and compared to the control mycelia cultured without induction with oxalic acid. Figure 5 shows the oxalate oxidase activity detected in the mycelia of the tested fungi. The highest activity was detected in the induced mycelium of *L. sulphureus* (166 U/mL). This activity was 22 times higher in comparison to the non-induced mycelium of this fungus. Similar induction of OXO activity after oxalic acid addition was observed in *A. biennis*, where it reached 30 U/mL and was 8.5 times higher than in the non-induced mycelium. The exception from this observation was *C. subvermispora*, which demonstrated higher OXO activity in the mycelia from the oxalic acid non-amended cultures. The rest of the tested mycelia exhibited higher OXO activity in the oxalic acid-amended cultures but this induction was not significant.

## Discussion

The generally accepted division assumes that fungi degrade oxalic acid via oxalic acid decarboxylase activity (ODC) (Sverduzić et al. 2005). The known fungal ODC are intracellular and have an inducible character (Mäkelä et al. 2002). In *Dichomitus squalens*, addition of oxalic acid stimulated oxalate decarboxylase activity but no increase in the transcript amount was detected, which suggests non-transcriptional upregulation of ODC (Mäkelä et al. 2009). The activities of oxalate degrading enzymes can be stimulated not only by oxalic acid but also by lowering the pH value of fungal cultures. This was demonstrated in *D. squalens* in the case of ODC and in *A. biennis* regarding OXO (Hu and Guo 2009; Mäkelä et al. 2014; Grąz et al. 2016). The oxidative pathway of oxalate degradation via OXO activity has been described predominantly in plants. It has been detected in barley (Kotsira and Clonis 1997; Requena and Bornemann 1999), wheat (Hu and Guo 2009), oats, rice, and rye (Lane 2000), maize (Vuletic and Sukalovic 2000; Lane 2000), and beet leaves and stems (Varalakshmi and Richardson 1992). We were prompted to look for oxalate oxidase in fungi by the fact that the ability to degrade oxalate via the oxidative pathway has been proved so far only in *C. subvermispora* (Aguilar et al. 1999; Escutia, et al. 2005) and *A. biennis* (Grąz et al. 2009, 2016). It is worth noticing that the well-characterized OXO from *C. subvermispora* is classified as a bicupin protein similar to fungal ODC, and the known OXO originating from plants are monocupins (Dunwell et al. 2000; Escutia et al. 2005). We observed in this study that the induction of oxalate-degrading enzymes by oxalic acid addition can elevate the hydrogen peroxide concentration in the fungal secretomes. The increased level of hydrogen peroxide in the cultures may indicate the oxidative pathway for oxalic acid degradation via oxalate oxidase. The reactive oxygen species were formed in the proposed catalytic cycles of ODC and OXO (Just et al. 2004; Burrell et al. 2007; Pastore et al. 2021). The presence of the hydroperoxyl radical during the turnover of *Bacillus subtilis* ODC was observed by Twahir et al. (2015). Hydrogen peroxide is needed by basidiomycete fungi, as these organisms produce extracellular enzymes which require hydrogen peroxide as a co-substrate. These classes of enzymes involved directly in lignin decomposition include heme-containing peroxidases (POD), namely lignin (LiP), manganese (MnP), and versatile (VP) peroxidase, as well as heme-thiolate haloperoxidases (Janusz et al. 2017). Oxidases found in fungal secretomes may be a source of hydrogen peroxide needed for reactions catalyzed by peroxidases. Among them, glucose oxidase (EC 1.1.3.4), glyoxal oxidase (GLOX; EC 1.2.3.5), aryl alcohol oxidases (AAO; EC 1.1.3.7), pyranose 2-oxidase (POX; EC 1.1.3.10), and cellobiose dehydrogenase (CDH; EC 1.1.99.18) are very important and well establish (Janusz et al. 2017). We postulated for OXO from *A. biennis* such a role in fungal metabolism in our earlier study (Graz et al. 2016). Oxalic acid supplementation can stimulate the secretion of MnP in *C. subvermispora* (Aguiar and Ferraz 2012). The stabilizing role of oxalic acid in the catalytic cycle of MnP is well known (Hofrichter 2002). In our earlier study, we reported changes in the gene expression level in *A. biennis* as a response to oxalic acid induction. This transcriptional study revealed that the oxalic acid addition caused down-regulation of genes coding for lignolytic enzymes, especially genes encoding VP and, to a lesser extent, MnP. The up-regulation was determined for the gene for cellulolytic enzymes, especially endo-β-1,4-xylanase (Grąz et al. 2017). The detailed analyses of the fungal secretome carried out to determine the content of oxalic acid degradation products presented in this study revealed that fungi with a low concentration of hydrogen peroxide in their secretome after 24 h of induction with oxalic acid had an increased level of formic acid and a reduced concentration of oxalic acid. This was observed in the cultures of *T. versicolor*, *G. odoratum*, *P. betulinus*, *N. frowardii*, *B. fumosa*, *S. commune*, *A. aegerita*, *F. fomentarius*, and *T. sanguinea*. This pattern of the secretome allows a conclusion that these fungi metabolized oxalates via oxalate decarboxylase. A similar drop in the oxalic acid concentration was also found in the secretomes of fungi classified to the group decomposing oxalate in the oxidative manner via oxalate oxidase, with the detectable increase in the hydrogen peroxide level in the media. A different situation was observed in the third group of the tested fungi, which demonstrated no increase in the content of hydrogen peroxide or formate in their secretomes and yet a decrease in the concentration of oxalic acid or even little accumulation of oxalic acid were observed, as in the secretome of *P. ostreatus*. The observed drop in the oxalic acid concentration may be related to its precipitation because divalent metal oxalates are insoluble and were not detectable in the secretomes during the experiment. Oxalic acid has the ability to chelate metals but can also be a metal precipitant. The deposition of oxalate salt is well known, with calcium and lead oxalate as the least soluble salts (Gadd et al. 2014; Gadd 2021). Finally, we managed to confirm the intracellular OXO activity in nine fungal mycelia of *S. commune*, *T. hirsuta*, *G. trabeum*, *A. biennis*, *C. unicolor*, *C. mediosetigera*, *T. sanguinea*, *C. subvermispora*, and *L. sulphureus*. Among them, only two fungi, i.e. *C. subvermispora* and *A. biennis* were previously described in the literature as OXO producers. In this study, only *A. biennis* and *L. sulphureus* showed potent induction of OXO activity after the oxalic acid addition. Further studies are necessary to establish whether this induction in *L. sulphureus* was connected with the pH value decline after the oxalic acid addition, as in the case of *A. biennis* (Grąz et al. 2016) or whether it was due to the substrate induction. The similarity of the catalytic mechanisms proposed for OXO and ODC is known (Just et al. 2004). OXO isolated from *C. subvermispora* exhibited a low rate of decarboxylase activity (Escutia et al. 2005). In the case of *B. subtilis,* it has been demonstrated that ODC activity can be converted into OXO activity by mutation in the active site of the enzyme (Burrell et al. 2007). To the best of our knowledge, there are some protein sequences for OXO in the NCBI database described for basidiomycete fungi, but there are no literature reports on oxalate oxidase activities in basidiomycete fungi, except for the *C. subvermispora* and *A. biennis* fungi mentioned above. This study provides the first description of the OXO activities in the analyzed fungi.

## Conclusions

As shown in the present study, the analyzed fungi can potentially decompose oxalic acid via the oxidative pathway, leading to the generation of hydrogen peroxide. We detected the OXO activity in nine basidiomycete fungi. It is necessary to further study the mechanism of the catalytic action of these enzymes and their structure. The elucidation of the regulation of the oxalate content and potential application of enzymes in the removal of excess of oxalates may be a promising tool in agriculture and medicine. Effective methods for oxalate removal can improve the nutritional value of forage plants and protect plants against fungal pathogens. Enzymes with new biochemical properties can be used in the diagnosis and prevention of kidney diseases caused by the precipitation of calcium oxalates.

## Data Availability

The datasets generated during the current study are available from the corresponding author on reasonable request.
